# Exploring single cell microbial protein as a sustainable fishmeal alternative in yellowtail kingfish (*Seriola lalandi*) diets: impacts on health and gut microbiome

**DOI:** 10.1186/s40104-024-01146-w

**Published:** 2025-02-02

**Authors:** Luke Pilmer, Lindsey Woolley, Alan Lymbery, Chinh Dam, Abigail Elizur, Md Javed Foysal, Gavin Partridge

**Affiliations:** 1 Centre for Sustainable Aquatic Ecosystems, Harry Butler Institute, Murdoch University, Murdoch, WA 6150 Australia; 2 Department of Primary Industries and Regional Development, Fleet Street, Fremantle, WA 6160 Australia; 3 Bioinovation Centre, University of the Sunshine Coast, Maroochydore, QLD 4558 Australia; 4 Research Institute for Aquaculture No.1, Dinh Bang, Tu Son, Bac Ninh, Vietnam; 5 School of Environmental and Life Sciences, The University of Newcastle, Callaghan, NSW Australia; 6 Oceans Institute, University of Western Australia, Stirling Highway, WA 6009 Australia

**Keywords:** Alternative proteins, Gene expression, Methanotrophic bacteria, Microbiome, Yellowtail kingfish

## Abstract

**Background:**

With the global expansion of aquaculture and the increasing demand for fish meal, identifying appropriate and sustainable alternative protein sources for aquafeeds has become essential. Single-cell protein (SCP), derived from methanotrophic bacteria, presents a promising alternative by converting methane into protein, potentially addressing both the need for alternative protein sources and reducing industrial greenhouse gas emissions. This study aimed to evaluate the effects of different levels of SCP inclusion (0%, 25%, 50%, and 75% fish meal replacement) on the health, gene expression, and gut microbiome of yellowtail kingfish (YTK, *Seriola lalandi*) following a 35-day growth trial.

**Results:**

The study found that SCP inclusion at the highest level of fishmeal replacement (75%) induced a mild inflammatory response in the hindgut of the fish. However, micromorphological assessments of the hindgut, serum biochemistry, and gene expression analyses revealed no significant detrimental effects from SCP replacement. Notably, there were indications of improved lipid digestibility with SCP. Furthermore, SCP inclusion significantly enhanced microbial richness and altered the composition of the gut microbiome, introducing beneficial bacterial taxa that may contribute to improved gut health and resilience.

**Conclusions:**

This study highlights SCP as a viable and sustainable alternative to fish meal in YTK diets. The findings suggest that SCP can be included in YTK diets without adverse health effects at moderate levels and may even offer benefits in terms of lipid digestibility and gut microbiome diversity. These results contribute to the advancement of more sustainable aquaculture practices.

**Supplementary Information:**

The online version contains supplementary material available at 10.1186/s40104-024-01146-w.

## Introduction

*Seriola lalandi,* commonly known as yellowtail kingfish (YTK), is a temperate, carnivorous, marine species, that is popular in the global sashimi market [1]. It is well-suited to aquaculture as it commands a high market price and is fast-growing [2]. YTK culture is an established industry in Australia and Japan [3, 4] and emerging in New Zealand, South Africa, Europe, and the Americas [5, 6]. Currently, commercial YTK diets require large amounts of fishmeal to optimise the health and growth of the fish, with most commercial diets for the species incorporating approximately 40% fishmeal to meet nutritional needs [7].

The rapid growth of the aquaculture industry and an increasing emphasis on environmental sustainability has encouraged researchers to investigate alternative sources of protein that can completely or partially replace fishmeal from the diets of cultured fish [8–14]. Previously studied alternative sources of protein include plants and legumes, animal by-products, algae, insects and single-cell proteins (SCP) [10, 15–18]. Of these, SCP derived from methanotrophic bacteria are of particular interest due to their ability to convert methane into protein. Methanotrophic bacteria utilize methane as a carbon and energy source and ammonia as a nitrogen source [19]. With increasing global concerns around industrial greenhouse gas emissions, the ability to turn waste methane into a product has both economic and environmental benefits [20].

Recent investigations have highlighted the effectiveness of methanotrophic bacterial meal, a promising alternative to traditional fishmeal, in the diets of various aquaculture species. Studies have shown positive outcomes for growth and digestibility in species such as Pacific white shrimp (*Litopenaeus vannamei*), Atlantic halibut (*Hippoglossus hippoglossus*), Atlantic salmon (*Salmo salar*), red sea bream (*Pagrus major*), rainbow trout (*Oncorhynchus mykiss*), and Japanese yellowtail (*Seriola quinqueradiata*) [9, 13, 14, 21–25]. In a previous study [7], we explored SCP inclusion at four dietary levels in YTK diets of 10%, 20% and 30% equating to 0%, 25%, 50%, and 75% fish meal replacement from a control diet containing 40% fish meal. In this 35-day trial, growth rates and feed conversion ratio (FCR) of fish were significantly improved in the diets that replaced 25% of fishmeal with SCP compared to the control. A consistent FCR, protein retention ability, and digestibility coefficients were obtained even with higher SCP replacement levels, despite reduced feed consumption and subsequently growth rates, indicating a potential for higher SCP replacements in YTK diets if palatability issues can be overcome.

While previous studies on methanotrophic bacterial meals have focused primarily on growth metrics, there has been limited exploration into the broader health impacts of dietary SCP inclusion, particularly concerning gene expression and microbiome composition in YTK. Emerging research suggests that dietary interventions can significantly influence gut microbiota, which in turn affects nutrient absorption, immune function, and overall fish health [26, 27]. Additionally, alterations in gene expression related to immune responses, metabolism, and stress have been observed in fish subjected to varying dietary compositions, including those containing alternative protein sources [28]. The YTK microbiome, which plays a crucial role in digestion and pathogen resistance, may be particularly sensitive to changes in diet composition, such as the replacement of fishmeal with SCP [29, 30].

This study aimed to examine the effects of SCP dietary replacement levels on a range of health and immune parameters in YTK, including changes in gene expression and microbiome composition. We hypothesised that there would be no adverse health impacts in fish fed for 35 d with SCP replacing 25%, 50%, and 75% of fishmeal, compared to a control diet containing 40% fishmeal.

## Methods

### Experimental design

This study used samples from fish in the palatability and growth experiment reported in Pilmer er al. [7]. Detailed experimental protocols are described therein. Briefly, 312 juvenile YTK, averaging 169.3 ± 0.6 g in weight, were individually tagged with RFID pit tags, then evenly and randomly distributed among 24 tanks, comprising 8 treatments (four levels of SCP inclusion, either with or without dietary additives, such as liquid tuna hydrolysate and garlic powder) with 3 replicate tanks per treatment. The SCP used was derived from methanotrophic bacteria (StringBio Pty Ltd., Bengaluru, India) provided by Ridley AgriProducts Pty Ltd. (Melbourne, Australia). All diets were formulated to be isonitrogenous (50% crude protein) and isolipidic (19% crude fat). The four levels of SCP inclusions were 0%, 10%, 20%, and 30% of the diet, which corresponded to 0%, 25%, 50%, and 75% fish meal replacement, respectively. The formulated diets were processed into 5 mm pellets using a commercial extrusion system with vacuum oil coating. During the 35-day growth trial, the fish were fed the experimental diets twice daily until satiety, the tanks were supplied with oxygenated seawater at a flow rate of 6 L/min, with ambient temperature maintained at 20 ± 1 °C. At the end of the trial, individual body weight was recorded for all fish and three fish per tank were then sampled for health measurements.

### Morphological and biochemical parameters

Whole blood was collected from the caudal vein using EDTA (2% solution; Sigma) coated needles and stored in 1-mL foetal tubes (K3E EDTA Mini collect®, Greiner Bio-One, Austria). Further samples of whole blood were collected without EDTA from the same fish and stored in 2-mL Eppendorf tubes for serum collection. The latter blood samples were allowed to clot at room temperature (~ 20 °C) for 1 h and then stored at 4 °C overnight before being spun for 10 min at 3,000 × *g* at 4 °C. Blood serum was removed, divided into triplicate 200 μL aliquots and frozen before analyses of biochemical parameters and serum antioxidant capacity. The biochemical parameters of cholesterol, alanine aminotransferase (ALT), glutamate dehydrogenase (GLDH), lipase, urea, total protein and triglyceride were measured on an ‘AU 680’ analyser. Fast, slow and total antioxidant capacity were measured using an e-BQC lab (BioQuoChem, Spain).

Samples of the liver, hindgut and pyloric caeca were dissected and fixed in 10% neutral buffered formalin for traditional histological examination. Histology sections were stained in H&E and PAS-Alcian Blue. Hindgut sections were used for the determination of lamina propria area as a percentage of villus area (% lamina propria) and muscular thickness. Further sections of the hindgut were dissected and immediately frozen in liquid nitrogen for analysis of myeloperoxidase (MPO) activity as a marker of neutrophil accumulation in the hindgut. The method used for MPO analysis was the same as described in Woolley et al. [31]. Briefly, frozen tissue was ground using a pre-chilled mortar and pestle and homogenised in a 0.5% hexadecyltrimethylammonium bromide (HTAB) solution prepared in 50 mmol/L phosphate-buffered saline (PBS). The homogenized samples were centrifuged, and the supernatant was collected. To determine MPO activity, a fluorescence-based assay was conducted: samples were diluted, and triplicates were loaded into a black 384-well microplate along with a working solution of 10 µmol/L 2-[6-(4-aminophenoxy)-3-oxo-3H-xanthen-9-yl] benzoic acid (APF) and 10 µmol/L hydrogen peroxide in PBS. Fluorescence was measured every minute for 30 min at an excitation wavelength of 485 nm and emission between 515–530 nm.

### Gene expression

Twelve genes involved in immune function and growth were targeted for analysis of expression in different tissues (Table 1). Total RNA was isolated from 30 mg samples of intestinal, liver, and brain tissues, all preserved in RNAlater. Samples from all tissues, were only analysed from fish in the SCP0%, SCP25%, and SCP75% treatment groups due to the lack of significant differences observed in other health parameters. These tissues were processed using a TissueRuptor (Qiagen, Melbourne, Victoria, Australia) in 600 µL of RLT buffer for thorough disruption and homogenization, followed by RNA purification with the RNeasy Mini Kit (Qiagen, Melbourne, Victoria, Australia), according to the manufacturer’s guidelines. The concentration of the extracted RNA was quantified using NanoDrop spectrophotometry (ND-2000), with detailed concentration data presented in Table S1. Subsequently, complementary DNA (cDNA) was synthesized from 2 µg of the total RNA via a reverse transcriptase reaction facilitated by the Tetro cDNA Synthesis Kit (Bioline, Australia), which includes a crucial step for the elimination of any potential genomic DNA contamination. Primers and probes specific to the genes under investigation (Table 1) were designed based on species-specific mRNA sequences utilizing the Roche Assay Design platform, as outlined in Table S2.

**Table 1:** Genes were analysed in the different tissues sampled for gene expression

Class	Gene	Abbreviation	Tissue
Immune	Interleukin 1	*itl1*	Liver
	Catalase	*cat*	Intestine, liver, brain
	Superoxide dismutase	*sod*	Intestine, liver, brain
	Hepcidin	*hep*	Liver
	Glutathione peroxidase	*gpx*	Intestine, liver, brain
Growth	Integumentary mucin	*i-mucin*	Intestine
	Mucin 2	*mucin-2*	Intestine
	Peptide YY	*yy*	Intestine, brain
	Cholecystokinin	*cck*	Intestine, brain
	Trypsin	*try*	Intestine
	Chymotrypsin	*chy*	Intestine
	Carboxypeptidase A	*cpa*	Intestine

Quantitative PCR (qPCR) assays were executed in duplicate on a Rotor-gene 6000 Thermocycler (Qiagen, Germany), employing 20 µL reaction mixtures that comprised the FastStart Universal Probe Master Mix (Rox; Roche Diagnostics GmbH), Universal Probe Library Probe (Roche), primers, molecular biology-grade water, and synthesised cDNA. Each assay included a non-template control for background amplification. Cycling conditions involved an initial uracil N-glycosylase pre-treatment, followed by a denaturation step and 35 amplification cycles, each with specific temperature and time settings.

For normalisation purposes, the stability of potential reference genes, including those coding for 18S rRNA, β-actin, and elongation factor 1 alpha, was evaluated, with the 18S rRNA gene being selected due to its consistent expression levels across samples. Data were normalised, log2 transformed and analysed using REST 2008 2.0.7 software and the GeneX tool for efficiency-corrected expression ratio calculations.

### Microbiome

Microbial DNA was isolated from the hindgut of experimental fish using the QIAamp 96 DNA QIACube HT kit (Cat. #51331, Qiagen, Germany), as per the manufacturer’s protocol. Intestinal tissue was removed before DNA extraction to minimize eukaryotic contamination. Whole tissue samples were preserved in RNAlater immediately after dissection. For microbial DNA extraction, 20 mg was digested with proteinase at 56 °C for an 1 h, followed by DNA extraction. The quantity of extracted DNA was determined through the Qubit dsDNA High Sensitivity assay (Cat. #Q32851, ThermoFisher Scientific) and then preserved at −20 °C until further processing. Prior to library construction, DNA samples were standardised to 10 ng/μL and re-measured using the Qubit dsDNA HS assay.

The V3–V4 region of the 16S rRNA gene, a highly conserved genetic marker widely used for bacterial identification and community profiling, was selectively amplified from the purified DNA using the Quick-16S Plus NGS Library Prep kit. The 16S rRNA gene was chosen because it allows for efficient characterisation of bacterial diversity and composition in complex microbiomes, such as the fish gut, making it an appropriate target for understanding microbial changes in response to dietary SCP. Amplification employed a single-step PCR with specific V3–V4 16S rRNA and Illumina i7 and i5 index primers, under defined thermocycling conditions. Post-amplification, products were electrophoretically assessed and, if necessary, subjected to additional amplification cycles. Purification of the correctly sized products (~ 600 bp) was achieved using MagBeads, with subsequent quantification and pooling following the kit’s guidelines. Paired-end sequencing was executed on the MiSeq platform at Genomics WA, Australia.

The ensuing Illumina reads underwent initial processing in qiime2, encompassing quality trimming, denoising, chimera filtering, and amplicon sequence variant (ASV) picking via the q2-dada2 plugin [32, 33]. This step included specific trimming parameters to eliminate low-quality sequences. Multiple sequence alignment and phylogenetic tree generation were conducted using Mafft and Fasttree, respectively [34, 35]. ASVs were classified at various taxonomic levels using a pre-trained naive Bayes classifier and the consensus blast method against the SILVA 138 database [36]. Taxonomy-based filtering was applied to remove sequences related to mitochondria and chloroplasts. For diversity and community composition analyses, samples were normalized to the lowest sequencing depth, and samples with fewer than 1,000 high-quality reads were excluded.

### Data analysis

The results were subjected to a comprehensive statistical analysis to ascertain the differences between treatments across various parameters, including growth immune and biochemical parameters, serum antioxidant capacity, and histomorphological measurements. Two-way ANOVA was used to examine the impacts of SCP levels and feed additives on immune and biochemical parameters, serum antioxidant capacity, and histomorphological measurements, using tank mean values (*n* = 3) as the unit of observation. When significant differences were identified, a post hoc Tukey’s test was performed to determine where these differences occurred. Proportional data were arcsine transformed and residual plots were examined to verify the assumption of normality in all analyses to account for the risk of type I errors due to multiple comparisons, the Benjamini–Hochberg procedure was employed, with significance threshold set at *P* < 0.05. All statistical analyses were performed using JMP (Version 15.0.0).

Microbial diversity analyses were conducted using the phyloseq, ape, and vegan (adonis) packages in R [37, 38]. Diversity within fish samples (alpha diversity) was estimated by ASV richness (number of ASVs) and the Shannon-Weiner diversity index that considers both ASV richness and evenness of abundances. Diversity indices were compared between treatment groups using a non-parametric two-way ANOVA (Scheirer–Ray–Hare test). Differences in microbial community composition between fish samples (beta diversity) were assessed using unweighted and weighted UniFrac distance metrics. The effect of SCP replacement level and feed additives on beta diversity was tested by a permutational analysis of variance (PERMANOVA, 1,000 permutations) applied to the distance matrices. Venn diagrams, generated with the MicroEco R package [39], illustrated the shared and unique ASVs among treatments, while taxa abundances were analysed and visualized using the phyloseq R package, focusing on taxa with over 1% read abundance. Differential abundances of higher taxa (genus and phylum-levels) among groups were identified using the MicrobiomeMarker R package [40], with ambiguous taxa categorized as “unclassified” for clarity in composition analysis.

## Results

### Histology

The lamina propria to villi ratio (LP/V) was significantly influenced by SCP replacement level (F = 9.248, *P* = 0.0064), but not by additives or the interaction of additives and replacement level (Table 2). The LP/V ratio increased with increasing SCP levels, being greatest in fish fed the SCP75% diet with additives (8.85% ± 0.21%) and lowest those fed the SCP25% diet without additives (6.66% ± 0.45%). No significant effects of SCP level, additives, or their interaction were observed on any of the other hindgut morphology measurements (Table 2).

**Table 2:** Histomorphology measurements in yellowtail kingfish after 35-day growth trial

Additive inclusion	Diet	Hindgut morphology (µm unless otherwise stated)																		LP/V ratio, %		
		**HC, mm**			**ME**			**MI**			**MU**			**S**			**TIW**					
Without additives	SCP0%	19.3 ± 0.5			78.6 ± 7.6			140.9 ± 18.0			209.7 ± 25.2			99.1 ± 8.1			339.2 ± 37.1			6.6 ± 0.3		
	SCP25%	16.9 ± 1.0			67.3 ± 6.2			144.1 ± 15.5			211.4 ± 19.2			106.5 ± 17.5			342.6 ± 36.0			6.7 ± 0.5		
	SCP50%	17.9 ± 0.3			75.6 ± 3.9			157.5 ± 12.6			233.4 ± 16.8			108.2 ± 8.9			378.4 ± 29.4			6.9 ± 0.7		
	SCP75%	18.1 ± 0.2			62.9 ± 2.8			137.1 ± 2.3			200.5 ± 3.4			98.9 ± 8.8			332.4 ± 7.6			7.7 ± 0.6		
With additives	SCP0%	18.2 ± 0.7			66.5 ± 9.5			136.5 ± 16.2			200.2 ± 25.0			95.2 ± 15.9			329.8 ± 42.5			7.5 ± 0.1		
	SCP25%	19.2 ± 1.1			59.8 ± 1.6			132.9 ± 4.4			192.8 ± 5.6			102.7 ± 4.6			328.5 ± 6.3			6.8 ± 0.3		
	SCP50%	19.0 ± 0.7			74.7 ± 2.3			144.8 ± 13.3			218.2 ± 14.0			103.8 ± 6.1			358.0 ± 23.3			7.7 ± 0.2		
	SCP75%	17.5 ± 0.4			67.2 ± 5.5			152.7 ± 9.7			221.8 ± 13.8			113.1 ± 25.8			375.4 ± 42.9			8.8 ± 0.2		
*P* Additives		0.463			0.336			0.709			0.664			0.949			0.992			0.028		
*P* SCP Replacement		0.325			0.558			0.418			0.427			0.485			0.338			**0.006**		
*P *Interaction		0.977			0.145			0.447			0.368			0.505			0.424			0.609		

Values are represented in mean ± SE (*n* = 3). *P* values were calculated using two-way ANOVA, with values in bold indicating a significant difference following the Benjamin-Hochberg procedure

*HC* Hindgut circumference, *ME* Muscularis externa, *MI* Muscularis interna, *MU* Muscularis thickness, *S* Submucosa, *TIW* Total intestinal wall, and *LP/V* Lamina propia area to villus area

### Biochemical parameters

There were significant effects of SCP replacement level on serum lipase, cholesterol, and triglycerides, with higher levels of lipase, but lower levels of cholesterol, and triglycerides as SCP replacement level increased (Table 3). There were no significant effects of dietary additives or an interaction between additives and SCP replacement level for any of these parameters. Serum urea levels were also influenced by SCP replacement level, but with a significant interaction with additives; the increase in urea levels with SCP replacement level was greater when feed contained additives (Table 3). All other biochemical parameters and measures of total antioxidant capacity showed no significant effect of SCP replacement level, dietary additives, or their interaction.

**Table 3:** Biochemical analysis and total antioxidant capacity measured in serum using e-BQC in yellowtail kingfish after 35-day growth trial

Additive inclusion	Diet	Blood biochemistry																								Antioxidant capacity, µC								
		**AST, U/L**			**ALT, U/L**			**GLDH, U/L**			**Lipase, U/L**			**Urea, mmol/L**			**Cholesterol, mmol/L**			**Triglyceride, mmol/L**			**Total protien,g/L**			**Q1**			**Q2**			**QT**		
Without additives	SCP0%	100.2 ± 38.8			16.8 ± 2.2			36.5 ± 4.0			8.2 ± 0.2			5.5 ± 0.7			7.0 ± 0.4			1.9 ± 0.1			40.2 ± 1.2			4.6 ± 0.3			41.6 ± 4.8			46.1 ± 5.1		
	SCP25%	104.7 ± 51.1			13.2 ± 2.1			22.6 ± 2.9			8.8 ± 0.3			6.0 ± 0.7			7.3 ± 0.4			1.5 ± 0.1			42.7 ± 1.3			4.7 ± 0.4			40.8 ± 3.6			45.5 ± 3.8		
	SCP50%	71.5 ± 16.0			15.2 ± 2.6			35.9 ± 3.3			8.3 ± 0.2			5.5 ± 0.3			6.3 ± 0.3			1.3 ± 0.1			41.5 ± 1.7			4.0 ± 0.4			39.9 ± 2.1			44.0 ± 2.5		
	SCP75%	89.3 ± 14.6			16.8 ± 2.5			46.5 ± 13.2			8.8 ± 0.2			5.5 ± 0.3			6.3 ± 0.4			1.3 ± 0.2			42.5 ± 1.5			5.1 ± 0.5			43.4 ± 1.5			48.5 ± 1.0		
With additives	SCP0%	61.3 ± 10.5			13.5 ± 1.5			32.3 ± 3.7			8.2 ± 0.2			4.2 ± 0.3			7.7 ± 0.4			1.7 ± 0.2			40.5 ± 1.7			3.9 ± 1.0			35.2 ± 1.6			39.1 ± 2.5		
	SCP25%	75.0 ± 19.1			13.2 ± 1.5			26.4 ± 2.6			8.5 ± 0.2			4.4 ± 0.3			8.2 ± 0.4			1.5 ± 0.1			41.3 ± 1.2			4.0 ± 0.6			40.4 ± 0.8			44.4 ± 1.2		
	SCP50%	124.2 ± 31.8			16.2 ± 3.2			28.4 ± 3.9			9.0 ± 0.0			6.1 ± 0.3			5.4 ± 0.1			1.0 ± 0.1			42.7 ± 0.5			3.4 ± 0.8			34.2 ± 5.4			37.6 ± 6.2		
	SCP75%	94.0 ± 11.3			17.7 ± 1.4			25.6 ± 5.1			9.0 ± 0.3			6.2 ± 0.5			6.0 ± 0.4			1.1 ± 0.1			42.3 ± 1.1			4.6 ± 1.2			40.2 ± 2.0			44.9 ± 2.5		
*P *Additives		0.882			0.824			0.076			0.434			0.140			0.746			0.175			1			0.238			0.098			0.089		
*P *SCP Replacement		0.932			0.406			0.185			**0.025**			**0.040**			** < 0.001**			**0.001**			0.225			0.474			0.452			0.391		
*P *Interaction		0.326			0.775			0.182			0.190			**0.007**			0.137			0.179			0.684			0.997			0.774			0.827		

Values are mean ± SE (*n* = 3).* P* values were calculated using two-way ANOVA, with values in bold indicating a significant difference following the Benjamin-Hochberg procedure. *AST* Aspartate aminotransferase, *ALT* Alanine aminotransferase, *GLDH* Glutamate dehydrogenase, *Q1* Fast antioxidants, *Q2* Slow antioxidants, *QT* Total antioxidants

###  Myeloperoxidase (MPO) activity

**Fig. 1:**
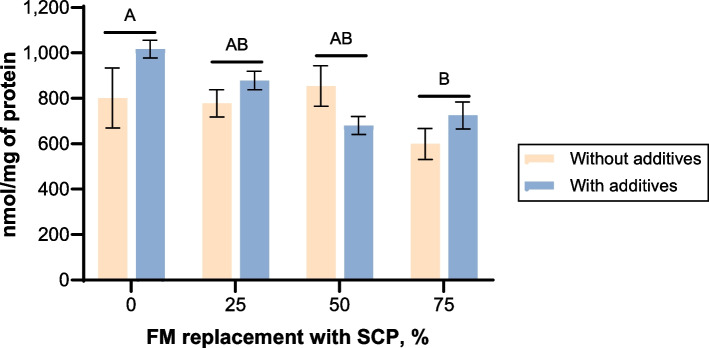
Myeloperoxidase activity in yellowtail kingfish hindgut after 35-day growth trial. Values are mean, with SE bars (*n* = 3). Different letters indicate significant differences between FM replacement levels, as determined by post hoc Tukey’s test

MPO in the hindgut of the fish was significantly affected by SCP replacement level (F = 4.121, *P* = 0.024), with MPO activity decreasing as SCP levels increased (Fig. 1). There was no significant effect of dietary additives on MPO or an interaction of additives and SCP replacement level.

### Gene expression

There were no significant effects of SCP replacement level or dietary additives, and no significant interaction between these terms, on gene expression levels in the intestine (Table 4) or brain (Table 5).

**Table 4:** Gene expressionm in the gut of yellowtail kingfish after 35-day growth trial

**Additive inclusion**	**Diet**	Digestive enzyme and hormone-encoded genes															Immune-related genes						Oxidative stress-related genes								
		***chy***			***try***			***cpa***			***cck***			***yy***			***mucin-2***			***i-mucin***			***gpx***			***cat***			***sod***		
Without additives	SCP0%	3.3 ± 0.1			3.8 ± 0.1			3.3 ± 0.1			3.5 ± 0.1			3.6 ± 0.2			3.6 ± 0.0			3.3 ± 0.2			3.9 ± 0.2			3.8 ± 0.0			3.7 ± 0.0		
With additives	SCP25%	3.5 ± 0.2			3.8 ± 0.1			3.5 ± 0.1			3.7 ± 0.1			4.0 ± 0.1			3.6 ± 0.1			3.7 ± 0.1			3.9 ± 0.0			3.9 ± 0.1			3.9 ± 0.1		
	SCP75%	3.5 ± 0.1			3.7 ± 0.1			3.4 ± 0.1			3.8 ± 0.0			3.7 ± 0.1			3.7 ± 0.1			3.6 ± 0.1			3.7 ± 0.1			3.9 ± 0.1			3.7 ± 0.0		
	SCP0%	3.2 ± 0.2			3.9 ± 0.1			3.3 ± 0.1			3.4 ± 0.1			3.4 ± 0.2			3.7 ± 0.0			3.4 ± 0.1			3.9 ± 0.0			3.8 ± 0.0			3.7 ± 0.0		
	SCP25%	3.5 ± 0.1			3.7 ± 0.1			3.5 ± 0.1			3.8 ± 0.0			3.9 ± 0.1			3.9 ± 0.0			3.5 ± 0.0			3.8 ± 0.1			3.9 ± 0.0			3.9 ± 0.1		
	SCP75%	3.2 ± 0.1			3.9 ± 0.1			3.5 ± 0.0			3.5 ± 0.1			3.9 ± 0.0			3.7 ± 0.1			3.7 ± 0.0			3.7 ± 0.1			3.8 ± 0.0			3.7 ± 0.0		
*P *Additives		0.283			0.321			0.957			0.163			0.696			0.057			0.777			0.636			0.338			0.833		
*P *SCP Replacement		0.266			0.365			0.037			0.042			0.022			0.077			0.041			0.088			0.114			0.067		
*P *Interaction		0.461			0.192			0.529			0.173			0.311			0.038			0.573			0.712			0.147			0.910		

Values are in mean relative expression level ± SE (*n* = 3).* P* values were calculated using two-way ANOVA, with no significant effects following the Benjamin-Hochberg procedure *chy* Chymotrypsin, *try* Trypsin, *cpa* Carboxypeptidase A, *cck* Cholecystokinin, *yy* Peptide YY, *i-mucin* Integumentary mucin, *gpx* Glutathione peroxidase, *sod* Superoxide dismutase, *cat* Catalase

**Table 5:** Gene expression in the brain of yellowtail kingfish after 35-day growth trial

**Additive inclusion**	**Diet**	Brain gene expression														
		***yy***			***cck***			***gpx***			***sod***			***cat***		
Without additives	SCP0%	3.9 ± 0.0			3.9 ± 0.0			3.6 ± 0.2			2.8 ± 0.2			3.9 ± 0.1		
With additives	SCP25%	4.1 ± 0.1			3.8 ± 0.0			3.7 ± 0.1			3.1 ± 0.2			3.9 ± 0.1		
	SCP75%	4.1 ± 0.1			3.8 ± 0.1			3.7 ± 0.1			2.9 ± 0.1			4.1 ± 0.1		
	SCP0%	4.0 ± 0.1			3.9 ± 0.0			3.5 ± 0.2			2.6 ± 0.1			3.7 ± 0.1		
	SCP25%	4.1 ± 0.0			4.0 ± 0.0			3.9 ± 0.1			3.4 ± 0.1			4.0 ± 0.1		
	SCP75%	4.1 ± 0.0			3.8 ± 0.1			3.8 ± 0.0			3.1 ± 0.1			3.8 ± 0.1		
*P *Additives		0.584			0.321			0.703			0.535			0.189		
*P *SCP Replacement		0.117			0.075			0.150			0.522			0.298		
*P *Interaction		0.806			0.984			0.430			0.403			0.482		

Values are in mean relative expression level ± SE (*n* = 3).* P* values were calculated using two-way ANOVA, with no significant effects following the Benjamin-Hochberg procedure. *yy* Peptide YY, *cck* Cholecystokinin, *gpx* Glutathione peroxidase, *sod* Superoxide dismutase, *cat* Catalase

In the liver, however, there was a significant interaction effect between additives and SCP replacement level on the expression of glutathione peroxidase (*gpx*; F = 13.14, *P* = 0.003), with expression increasing with SCP replacement levels when there were no additives in the diet, but not when additives were present (Table 6). Interleukin-1 (*itl1*) expression in the liver showed significant effects of SCP replacement level (F = 121.34, *P* ≤ 0.0001), additives (F = 17.41, *P* ≤ 0.001), and their interaction (F = 10.13, *P* = 0.007). Expression increased when additives were added at 0% replacement level, but not at 25% and 75% replacement levels, and increased with increasing replacement level, although this was more marked with dietary additives (Table 6). There were no significant effects of additive inclusion, SCP replacement level, or their interaction on the expression of superoxide dismutase (*sod*), catalase (*cat*), and hepcidin (*hep*)*.*

**Table 6:** Gene expression in the liver of yellowtail kingfish after 35-day growth trial

**Additive inclusion**	**Diet**	Oxidative stress-related genes									Immune-related genes					
		***gpx***			***sod***			***cat***			***hep***			***itl1***		
Without additives	SCP 0%	3.2 ± 0.2			3.6 ± 0.0			3.9 ± 0.0			3.5 ± 0.1			2.9 ± 0.0		
With additives	SCP25%	3.4 ± 0.0			3.8 ± 0.0			3.8 ± 0.0			2.9 ± 0.1			3.2 ± 0.0		
	SCP75%	3.6 ± 0.0			3.9 ± 0.0			3.8 ± 0.0			3.1 ± 0.1			3.6 ± 0.1		
	SCP 0%	3.6 ± 0.0			3.8 ± 0.1			3.9 ± 0.0			3.4 ± 0.1			3.3 ± 0.0		
	SCP25%	3.5 ± 0.0			3.8 ± 0.1			3.8 ± 0.1			3.5 ± 0.2			3.3 ± 0.1		
	SCP75%	3.4 ± 0.1			3.8 ± 0.1			3.8 ± 0.0			3.3 ± 0.2			3.6 ± 0.0		
*P *Additives		0.122			0.558			0.993			0.118			** < 0.001**		
*P *SCP Replacement		0.092			0.023			0.069			0.173			** < 0.0001**		
*P *Interaction		**0.003**			0.070			0.892			0.405			**0.007**		

Values are in mean relative expression level ± SE (*n* = 3).* P* values were calculated using two-way ANOVA, with values in bold indicating a significant difference following the Benjamin-Hochberg procedure (*n* = 3). *gpx* Glutathione peroxidase 1, *sod* Superoxide dismutase, *cat* Catalase, *hep* Hepcidin, *itl1* Interleukin 1

### Microbiome

The analysis yielded over 1.4 million reads, with an average of approximately 21,928 high-quality, non-chimeric reads per sample, equating to 612 ASVs. After excluding singletons, a mean ASV frequency of over 20,000 per sample was obtained. After removing sequences related to mitochondria and chloroplasts, 12 unique phyla, 44 orders, and 65 genera were identified.

Rarefaction curve analysis indicated adequate sampling depth for over 90% of the samples, with a notable exception in fish fed the SCP50% diet, where some samples exhibited elevated lines at the lowest sampling depth, suggesting potential unique ASVs left in the community representing more species richness (observed and Chao1) (Fig. S1). Similarly, samples with dietary additives also displayed upward trending rarefaction lines (Fig. S2).

**Fig. 2:**
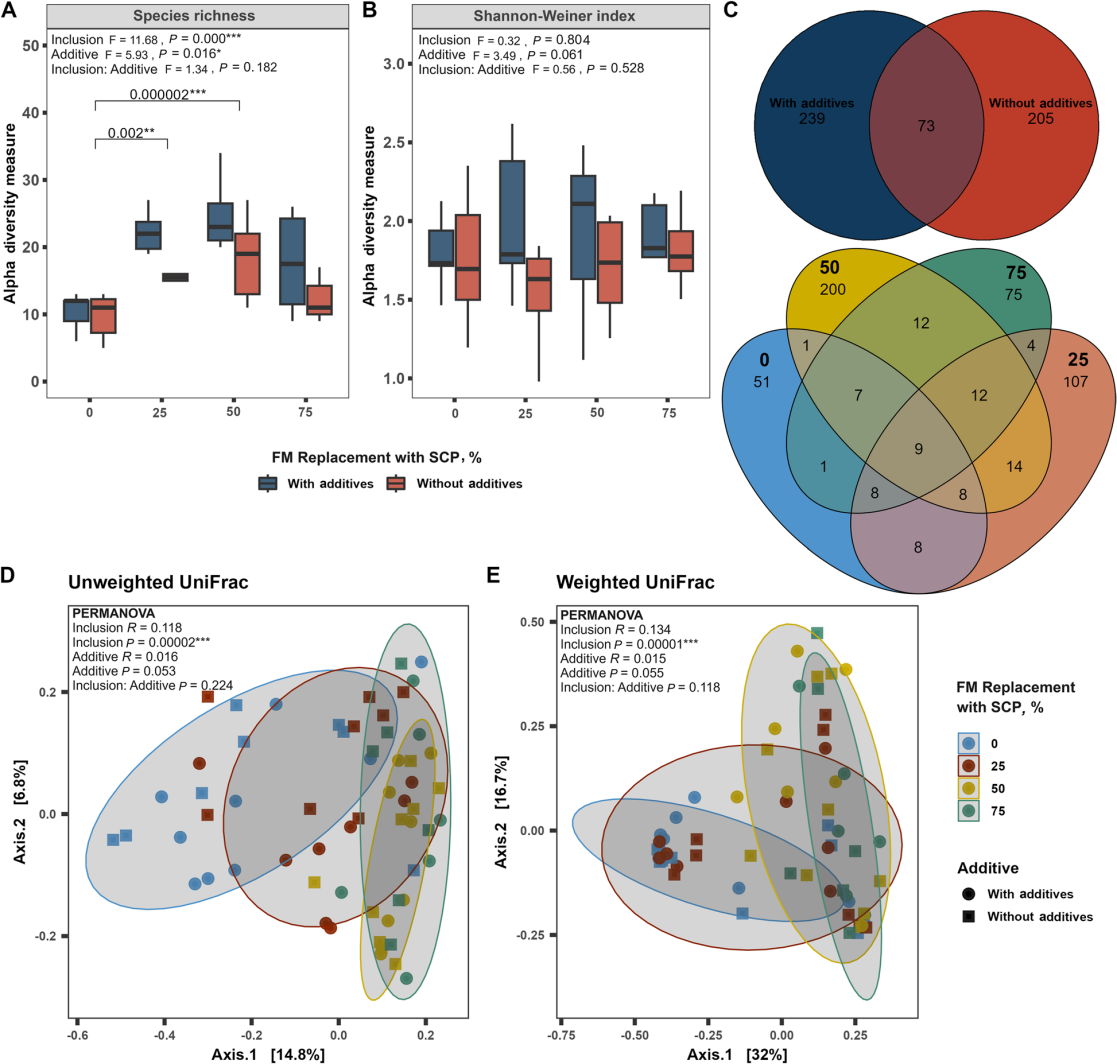
Alpha–beta diversity analysis of gut microbiota in yellowtail kingfish after 35-day growth trial. **A** and **B** Diversity of gut bacteria in terms of species richness and Shannon-Weiner index. Boxplots with different superscript letters at the top indicate significantly different mean values between additives (with and without) within a replacement level. **C** Number of shared and unique ASVs in fish-fed diets with and without additives (top), and at different SCP replacement levels (bottom). **D** and **E** PCoA plots representing beta-diversity for unweighted and weighted UniFrac distance metric

There were significant effects of both SCP replacement level (*P* < 0.001) and dietary additives (*P* = 0.016) on ASV richness, with no significant interaction. Richness was greater in fish that received dietary additives, and at intermediate (25% and 50%) replacement levels (Fig. 2A). In contrast to ASV richness, there were no significant effects of SCP replacement level or dietary additives on the Shannon-Weiner diversity index (Fig. 2B).

Analysis of unshared ASVs revealed that 40%–46% of ASVs were unique to samples with or without additives, with only 14% shared between the two groups. The SCP50% replacement level contributed to approximately 40% of the unshared ASVs, followed by SCP 25% (21%), SCP 75% (14.5%), and SCP 0% (10%) (Fig. 2C).

Both unweighted (*R*^2^ = 0.118, *P* = 0.00002) and weighted (*R*^2^ = 0.118, *P* = 0.042) UniFrac analyses demonstrated significant differences in microbial community composition among SCP replacement levels (*P* < 0.001), with no effects of dietary additives or the interaction between SCP replacement level and additives (Fig. 2D and E).

**Fig. 3:**
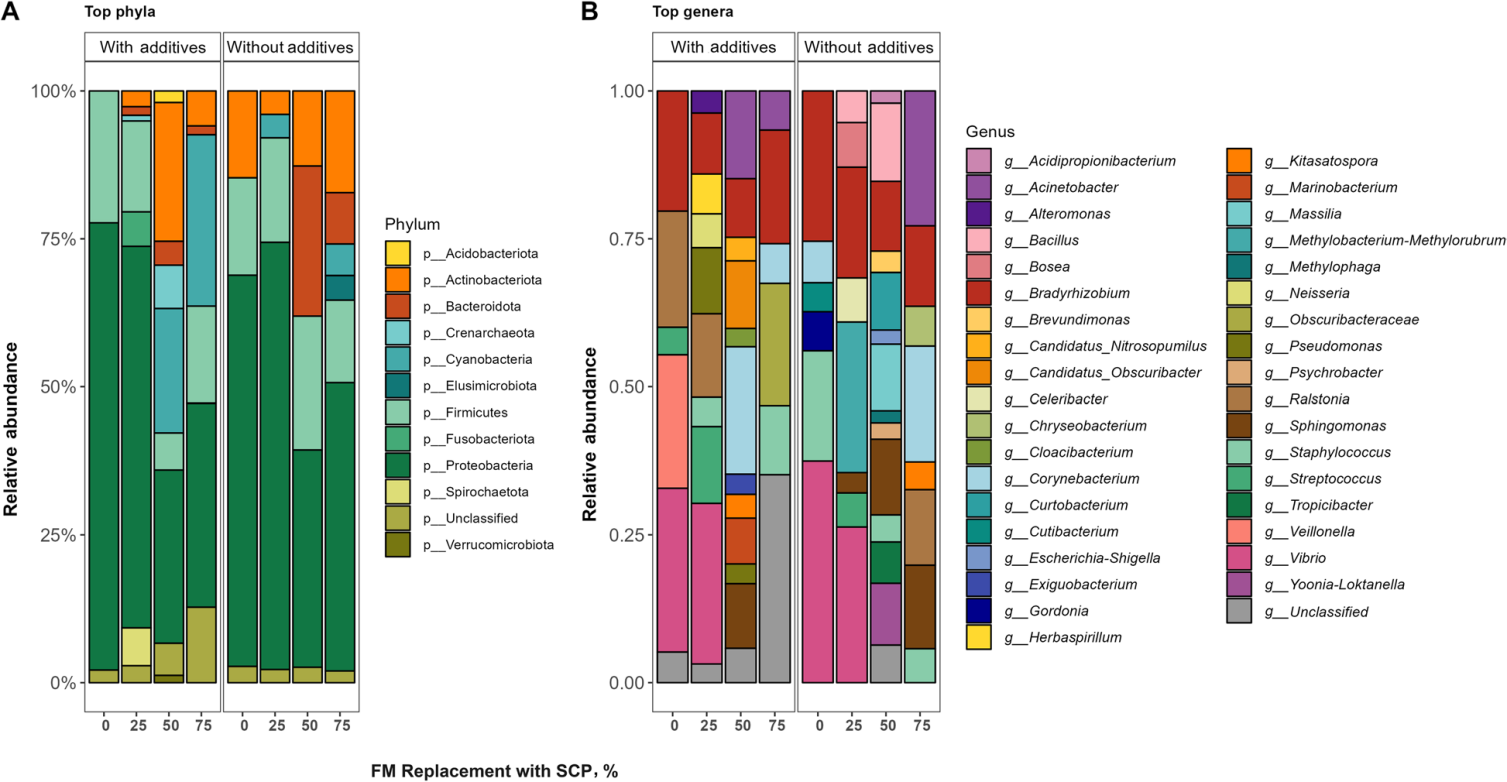
Relative abundance of bacteria at phylum (**A**) and genus (**B**) level in the gut of yellowtail kingfish after 35-day growth trial. Phyla and genera with more than 1% relative abundance were considered for plotting

Analysis of the relative abundance of bacterial phyla revealed that Proteobacteria-dominated bacterial communities were observed across all samples, regardless of SCP replacement levels and additives. In the 0% and 25% SCP diets, Proteobacteria and Firmicutes together comprised approximately 75% of the gut microbial communities. In contrast, fish from the 50% and 75% replacement levels exhibited a notable increase in Cyanobacteria abundance, accounting for 24% and 28% of the microbial reads, respectively, alongside a consistent presence of Proteobacteria at 21%. Actinobacteriota showed a high abundance (24%) in the 50% replacement level with additives, whereas other treatments displayed uniformly low Actinobacteriota abundance (5%–15%) without additives. Firmicutes abundance was lowest (5%) in the 50% replacement level with additives but ranged from 10%–18% in all other samples (Fig. 3A, Fig. S3).

At the genus level (Fig. 3B), *Vibrio* was more abundant (14% to 26%) in fish from the 0% and 25% replacement diet, while its presence was minimal (< 1%) in fish fed the higher replacement diets (SCP50% and SCP75%). *Bradyrhizobium* was consistently present across all samples, making it a ‘core’ component of the YTK gut microbiota. *Sphingomonas*, absent at 0% replacement level, was found in 5% to 12% of fish fed with higher SCP replacement diets. Notably, fish fed the 75% SCP diet had more opportunistic pathogens such as *Staphylococcus* and *Corynebacterium*. Additionally, 26% of bacteria in fish fed the 75% SCP replacement diet with additives remained unclassified, indicating a potential for undiscovered microbial diversity.

**Fig. 4:**
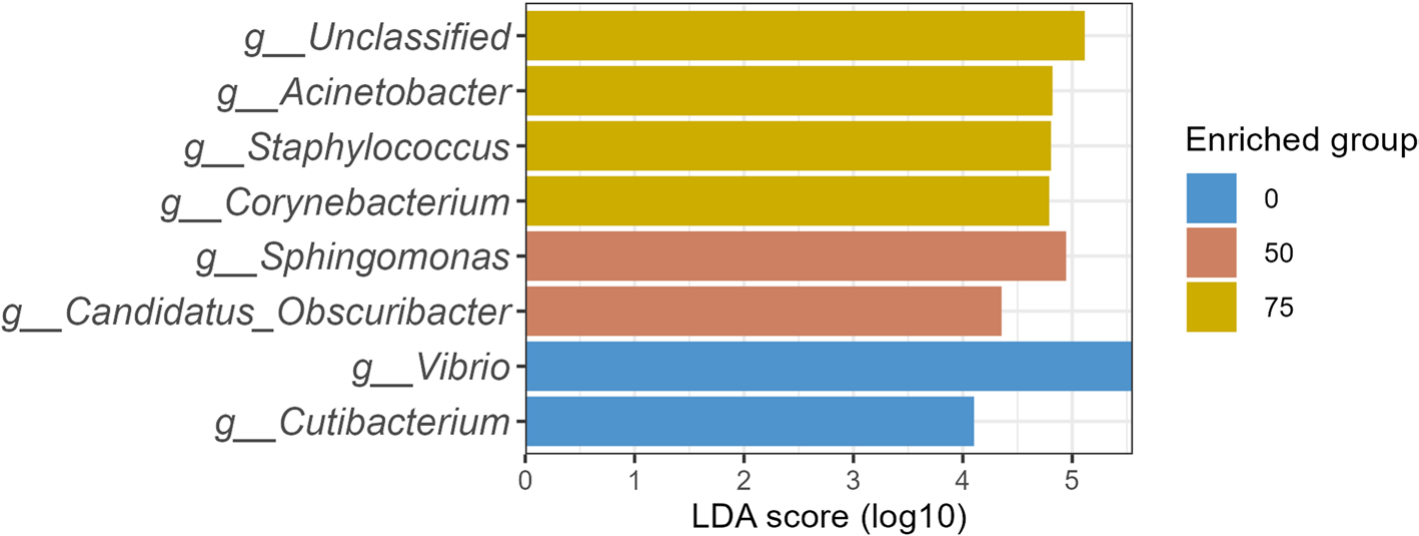
Differential abundance (DA) of gut microbiota in yellowtail kingfish after 35-day growth trial. An LDA score of 2.0 and above following the Kruskal–Wallis, with a Bonferroni adjustment *P*-value of < 0.05, was considered for DA analysis

Fish in the 50% SCP replacement diet had a more diverse gut microbiota compared to fish in other replacement levels. Differential abundance analysis highlighted a significantly higher abundance of *Vibrio* and *Cutibacterium* at 0% replacement, whereas *Sphingomonas* and *Candidatus obscuribacter* were more abundant in fish from the 50% SCP group (Fig. 4). Fish from the 75% SCP group showed significantly higher abundance of *Acinetobacter*, *Corynebacterium*, *Staphylococcus*, and several unclassified genera (Fig. 4). No specific genera were enriched in fish from the 25% SCP group, underscoring the nuanced impact of SCP levels on community composition.

## Discussion

As global aquaculture industries continue to grow, sustainable alternatives to fishmeal are continuously being explored. Single-cell microbial protein has shown promise as an alternative with its ability to capture and utilise greenhouse gases. Our previous research demonstrated that single-cell microbial protein (SCP), capable of capturing and utilizing greenhouse gases, serves as a promising alternative in the diet of YTK. In that study, fish fed a diet with 25% SCP replacement consumed less feed than those on the control diet but achieved comparable growth rates, leading to an improved feed conversion ratio (FCR). Higher levels of SCP replacement resulted in decreased feed consumption and growth rate, but FCR, protein retention efficiency, and apparent digestibility coefficients were maintained [7]. Building on these findings, the current study found no major negative health impacts, and increased gut microbial richness when juvenile YTK were fed diets substituting fishmeal with SCP at various levels.

### Health

Over the course of 35 d, this study observed no major adverse health effects from any of the diets, although there was some evidence for mild inflammation at the highest replacement level, as indicated by the lamina propria to villi area ratio, decreased myeloperoxidase (MPO) activity, and upregulation of some immune-related genes in the hindgut. The lamina propria to villi area ratio is crucial as it reflects the structural integrity and functionality of the gut, with a balanced ratio indicating effective nutrient absorption and a healthy gut environment [41]. While the ratio was elevated at increased SCP replacement levels, it still fell within a healthy range for juvenile YTK [41]. Similar results were found in previous studies on Atlantic salmon [42], barramundi [31], and black seabream [43], where no morphological damage to the gut was observed with the use of SCP in these diets. Importantly, although this study was 35 d in duration, it is sufficient to detect early signs of gut inflammation, as previous studies have shown that acute enteritis can manifest between 34–42 d [44, 45]. This suggests that while no major adverse health impacts were observed, the timeframe of this study was adequate to capture early indicators of gut health issues.

MPO activity has been used to indicate neutrophil infiltration and subsequent inflammation within the lamina propria of YTK, with a decrease in MPO levels indicative of inflammation [46]. MPO activity is a key indicator of neutrophil infiltration and inflammation within the lamina propria in YTK. Stone et al. [44] demonstrated that a reduction in MPO levels was associated with a decrease in inflammation, highlighting its utility as a marker for assessing inflammatory responses in the gut. In the current study, fish fed with the highest replacement level of SCP had significantly less MPO activity compared to fish fed with no FM replacement, suggesting that although the lamina propria area was significantly higher in the diets containing SCP, the reduced MPO levels indicate that this was not indicative of enteritis.

Serum biochemistry is routinely used in diagnostics to determine the health of aquaculture fish species. This study found significant differences in the lipase, cholesterol, and triglycerides in fish fed with SCP, but no significant changes in any of the other measured parameters. The significant rise in lipase levels in fish fed increasing levels of SCP and at the same time lower levels of triglycerides are similar to that found in barramundi fed the same SCP [31]. The reduction in serum cholesterol is likely linked to reducing dietary cholesterol as fishmeal inclusion decreased but could also be the result of improved lipid digestibility, consistent with the reduced triglycerides. Increases in urea are documented in both Atlantic salmon and barramundi fed SCP diets [22, 31] and these increased levels have been attributed to higher nucleic acids in the bacterial meal compared to fishmeal. Despite changes in these biochemical parameters with SCP inclusion, all biochemical indicators still fell within healthy ranges for YTK [47].

Although no differential gene expression was found in the intestine or brain of fish on the diets containing SCP, there were differences in expression levels of several genes related to oxidative stress and immune response in the liver. Specifically, glutathione peroxidase (*gpx*) showed a significant interaction between additives and SCP replacement, with the highest expression in fish fed the SCP75% diet without additives. This shows that high levels of SCP replacement can influence antioxidant enzyme activity and suggests that fish fed this diet were dealing with oxidative stress, consistent with the indications of inflammation in the hindgut. Interleukin-1 (*itl1*) expression was also significantly affected by SCP replacement and additives, with the highest expression in fish fed the SCP75% diet. *Itl1* was included as it is a pro-inflammatory cytokine that plays a crucial role in initiating immune responses to stress or injury, including dietary stress. Previous studies in YTK have shown that inflammation in the gut can occur when fishmeal is substituted with terrestrial alternatives, such as soy[44]. This suggests that SCP replacement may play a role in modulating immune responses, possibly contributing to the mild inflammatory effects observed in histomorphological analyses. In contrast, the expression of superoxide dismutase (*sod*), catalase (*cat*), and hepcidin (*hep*) did not show significant changes with SCP replacement, indicating that these aspects of the oxidative stress response and immune function were not markedly affected by the dietary treatments [48].

### Microbiome

Microbiome analysis of YTK gut found an increase in bacterial ASV richness and a change in ASV composition with SCP replacement in the diet, and this was most marked at replacement levels of 25% and 50%. This finding underscores the potential of a moderate level of SCP replacement to introduce beneficial new bacterial taxa to the gut microbiome, which could positively affect the host’s nutrient metabolism and immune function. Similar to how Aguilera et al. [49] observed shifts in microbial communities in response to fish growth stages, SCP inclusion may introduce beneficial new taxa that impact nutrient metabolism and immune responses in yellowtail kingfish. This diversification is particularly encouraging, given that research on cage-cultured YTK by Legrand et al. [50] highlighted a diminished microbial diversity in fish afflicted with enteritis, underscoring the potential health benefits of a richer gut microbiome. Despite the increased species richness, the lack of significant difference in the Shannon-Weiner diversity index suggests that the overall distribution of these species remains relatively uniform, which may indicate a resilient core microbiome that adapts to changes in SCP replacement levels while maintaining a balanced gut ecosystem.

The dominance of Proteobacteria across all samples is consistent with previous studies on aquatic species, indicating a stable presence of this phylum in the gut environment [51]. This aligns with the findings of Aguilera et al. [49], where Proteobacteria, particularly genera like *Pseudomonas*, were predominant in juvenile yellowtail kingfish. The consistent detection of *Bradyrhizobium* as a ‘core’ member of the gut microbiota in all samples underscores its likely contribution to the gut ecosystem’s stability and functionality in YTK. The association of *Bradyrhizobium* with lupin meal in the diet suggests that dietary components can have a direct influence on gut microbial composition. [52]. Despite these consistencies, the beta-diversity analysis revealed significant impacts of SCP replacement on the gut microbiota, with both presence-absence and relative abundance of bacterial taxa being affected, which could have implications for the host’s health. Notably, the reduction in *Vibrio* spp. in diets with higher SCP replacement is particularly encouraging, as *Vibrio* spp. are often associated with pathogenic outbreaks in aquaculture [53]. This reduction could signify a shift towards a microbial community composition that supports fish health and reduces disease risk. As highlighted in Aguilera et al. [49], *Vibrio* was one of the dominant bacterial genera in the gut of juvenile yellowtail kingfish. The reduction of this genus with SCP inclusion in our study may signify a shift toward a healthier gut microbiota. However, the increase in cyanobacterial abundance with higher SCP levels may have implications for gut health and nutrient absorption, warranting further investigation. In addition, the occurrence of opportunistic pathogens such as *Staphylococcus* and *Corynebacterium* in fish fed the SCP 75% diet raises concerns that elevated SCP levels might disturb the microbial equilibrium, potentially fostering conditions favourable for pathogen colonization. This aligns with the findings of Aguilera et al. [49], who observed *Staphylococcus* as a predominant genus in the early growth stages of yellowtail kingfish, suggesting that high SCP inclusion might create conditions conducive to the growth of opportunistic pathogens. Romarheim et al. [54] found that large and/or water-insoluble components found in *M. capsulatus* are important for maintaining the normal intestinal homeostasis in fish, further supporting SCP’s potential in gut health management. The benefits of increased microbiome diversity, such as enhanced nutrient metabolism, improved immune function, and protection against pathogens, underscore the importance of balancing SCP inclusion to optimize gut health. Greater microbial diversity helps create a stable gut environment that supports nutrient absorption and resilience to dietary changes [55]. These findings emphasize the nuanced influence of SCP replacement on the gut microbial ecosystem and highlight the complex balance between promoting beneficial microbial diversity and managing the risk of opportunistic pathogens.

Nevertheless, the differential abundance of bacterial genera across different SCP replacement levels and additives suggests that dietary manipulation can be a powerful tool in shaping the gut microbiome. The increased diversity observed in the SCP25% and SCP50% diets could be beneficial for nutrient absorption and immune modulation. Stone et al. [47] found that disease symptoms such as enteritis are associated with decreased microbial richness, diversity, and evenness, alongside a dominance of certain bacterial taxa in YTK. This parallels findings from a study in gilthead sea bream (*Sparus aurata*) that demonstrated increased gut microbiome diversity with 20% SCP replacements [56]. Notably, previous studies in YTK, which ran for 30–33 d, found significant shifts in microbial composition and function over similar durations to this study [49, 57]. This justifies the trial length, demonstrating that similar timeframes are adequate to capture meaningful changes in microbiome composition and associated functional outcomes. However, this benefit is juxtaposed with the potential risk of opportunistic pathogens at higher replacement levels, and balancing these factors is crucial for optimizing gut health and the overall well-being of the host species.

## Conclusion

This study employed a comprehensive approach, encompassing micromorphological assessments, serum biochemistry, and gene expression analyses, to investigate the effects of SCP on fish health. Notably, the microbiome analysis sheds light on SCP’s role in shaping gut microbial diversity and composition, which are pivotal for the overall health and growth of YTK. As the quest for sustainable feed alternatives in aquaculture continues, this research marks a significant step toward diminishing the dependence on traditional fishmeal, highlighting SCP’s potential as a promising option. This is particularly relevant when considering optimal replacement levels and the palatability of SCP. Despite the lack of observed clinical health impacts within the duration of this study, the complexity of fish nutrition and health warrants further, more detailed investigations. Future research should focus on unravelling the extensive physiological, immunological, and microbial dynamics associated with prolonged SCP usage, aiming for a comprehensive understanding of its implications for fish health and growth in the context of sustainable aquaculture.

## Supplementary Information

Supplementary Material 1: Table S1 RNA concentration (ng/µL). Table S2 Details of qPCR primers and probes. Fig. S1 Rarefaction curve indicating the depth and saturation level of samples with different SCP replacement levels. A stationary curve for bacterial diversity was observed for all samples except some for SCP 50. Fig. S2 Rarefaction curve indicating the depth and saturation level of samples with or without additives. A stationary curve for bacterial diversity was observed for all samples except for some samples with additives. Fig. S3 Replicate-based relative abundance of Kingfish gut bacteria at phylum level in different treatments after feeding trial.

## Data Availability

The datasets used and/or analysed during the current study are available from the corresponding author on reasonable request. The raw sequence data in fastq format have been deposited to National Centre for Biotechnology Information (NCBI) and currently available under the BioProject accession number PRJNA726745.

## Abbreviations

ALT: Alanine aminotransferase
APF: 2-[6-(4-Aminophenoxy)-3-oxo-3H-xanthen-9-yl] benzoic acid
ASV: Amplicon sequence variant
cat: Catalase
cDNA: Complementary DNA
CTAB: Cetyltrimethylammonium bromide
EDTA: Ethylenediaminetetraacetic acid
FCR: Feed conversion ratio
FM: Fish meal
GLDH: Glutamate dehydrogenase
gpx: Glutathione peroxidase
H&E: Hematoxylin and eosin
hep: Hepcidin
itl1: Interleukin-1
MPO: Myeloperoxidase
PAS: Periodic acid-Schiff
PBS: Phosphate buffered saline
PERMANOVA: Permutational multivariate analysis of variance
qPCR: Quantitative polymerase chain reaction
REST: Relative expression software tool
RFID: Radio frequency identification
RNA: Ribonucleic acid
rRNA: Ribosomal RNA
SCP: Single-cell protein
sod: Superoxide dismutase
YTK: Yellowtail kingfish
