# Supplementary material for: Exploring single cell microbial protein as a sustainable fishmeal alternative in yellowtail kingfish (Seriola lalandi) diets: impacts on health and gut microbiome
Source: J Anim Sci Biotechnol. 2025 Feb 2;16:16. doi: 10.1186/s40104-024-01146-w (PMC11787759; doi:10.1186/s40104-024-01146-w)
Supplement: Supplementary file 1 — Supplementary Material 1: Table S1 RNA concentration (ng/µL). Table S2 Details of qPCR primers and probes. Fig. S1 Rarefaction curve indicating the depth and saturation level of samples with different SCP replacement levels. A stationary curve for bacterial diversity was observed for all samples except some for SCP 50. Fig. S2 Rarefaction curve indicating the depth and saturation level of samples with or without additives. A stationary curve for bacterial diversity was observed for all samples except for some samples with additives. Fig. S3 Replicate-based relative abundance of Kingfish gut bacteria at phylum level in different treatments after feeding trial. [file 40104_2024_1146_MOESM1_ESM.docx]

**Supplementary Figures and tables**

Table S1. RNA concentration (ng/µL)

| Sample | fish | tank | tissue | RNA concentration ng/µL |
| --- | --- | --- | --- | --- |
| 1 | 1 | 1 | hindgut | 1500.5 |
| 2 | 1 | 9 | hindgut | 696 |
| 3 | 1 | 17 | hindgut | 1166 |
| 4 | 1 | 2 | hindgut | 1064.4 |
| 5 | 1 | 10 | hindgut | 354.8 |
| 6 | 1 | 18 | hindgut | 1023.6 |
| 7 | 1 | 3 | hindgut | 505 |
| 8 | 1 | 11 | hindgut | 634.9 |
| 9 | 1 | 19 | hindgut | 606.2 |
| 10 | 1 | 4 | hindgut | 680.2 |
| 11 | 1 | 12 | hindgut | 597.3 |
| 12 | 1 | 20 | hindgut | 535.7 |
| 13 | 1 | 7 | hindgut | 572.6 |
| 14 | 1 | 15 | hindgut | 566.2 |
| 15 | 1 | 23 | hindgut | 549.8 |
| 16 | 1 | 8 | hindgut | 539.1 |
| 17 | 1 | 16 | hindgut | 526.2 |
| 18 | 1 | 24 | hindgut | 580.9 |
| 19 | 1 | 1 | brain | 166.2 |
| 20 | 1 | 9 | brain | 149.8 |
| 21 | 1 | 17 | brain | 128 |
| 22 | 1 | 2 | brain | 124.2 |
| 23 | 1 | 10 | brain | 119.5 |
| 24 | 1 | 18 | brain | 160.5 |
| 25 | 1 | 3 | brain | 201.3 |
| 26 | 1 | 11 | brain | 181.7 |
| 27 | 1 | 19 | brain | 124.6 |
| 28 | 1 | 4 | brain | 110 |
| 29 | 1 | 12 | brain | 276.8 |
| 30 | 1 | 20 | brain | 112.3 |
| 31 | 1 | 7 | brain | 153.5 |
| 32 | 1 | 15 | brain | 109.4 |
| 33 | 1 | 23 | brain | 175.9 |
| 34 | 1 | 8 | brain | 116.8 |
| 35 | 1 | 16 | brain | 107.4 |
| 36 | 1 | 24 | brain | 658 |
| 37 | 1 | 1 | liver | 436.6 |
| 38 | 1 | 9 | liver | 420.8 |
| 39 | 1 | 17 | liver | 192.4 |
| 40 | 1 | 2 | liver | 606.2 |
| 41 | 1 | 10 | liver | 233.5 |
| 42 | 1 | 18 | liver | 173.9 |
| 43 | 1 | 3 | liver | 479 |
| 44 | 1 | 11 | liver | 338.2 |
| 45 | 1 | 19 | liver | 311.2 |
| 46 | 1 | 4 | liver | 981.8 |
| 47 | 1 | 12 | liver | 671.1 |
| 48 | 1 | 20 | liver | 1400 |
| 49 | 1 | 7 | liver | 298.5 |
| 50 | 1 | 15 | liver | 297.8 |
| 51 | 1 | 23 | liver | 457.4 |
| 52 | 1 | 8 | liver | 490.8 |
| 53 | 1 | 16 | liver | 445.7 |
| 54 | 1 | 24 | liver | 543 |

Table S2. Details of qPCR primers and probes

| Gene name | Sequences | Probes’s number |
| --- | --- | --- |
| elongation factor 1-α -F | GGATGGCATGGAGACAACAT | 54 |
| elongation factor 1-α- R | ATCTTCCATCCCTTGAACCA | 54 |
| 18S-F | AGGACTCCGGTTCTATTTTGTG | 57 |
| 18S-R | CGGCCGTCCCTCTTAATC | 57 |
| β-actin - F | CCTGTCCTGCTCACAGAGG | 7 |
| β-actin - R | TGTTGAAGGTCTCGAACATGA | 7 |
| interleukin 1 (itl1) -F | GCCAAACGCTACAGAACCTT | 42 |
| interleukin 1 (itl1) -R | CCACTTTGGGTGTCTTGGAC | 42 |
| i- mucin (i-muc) - F | TGTGCTCCTGGTTCGACTC | 157 |
| i- mucin (i-muc) - R | ACGGTGCAGGAGTACTTGAAA | 157 |
| mucin 2 (muc2) - F | CACCTGTGACCAGATGTTGC | 1 |
| mucin 2 (muc 2) - R | GTTCAGGGTCAATCAGTTTGG | 1 |
| superoxide dismutase (sod)- F | GCTCCTTCCAGAAAATGAAAGAG | 84 |
| superoxide dismutase (sod) - R | TCCACTCTGCTTGTCATAGCC | 84 |
| glutathione peroxidase (gpx) -F | CACGGGCCTTACCATAATCTT | 2 |
| glutathione peroxidase (gpx) -R | TCCAGGACGGACATACTTCA | 2 |
| peptite YY -F | GCAGAGCTCCTGTTTGGTG | 54 |
| peptite YY-R | CACCACATGTAGGAATCGTCA | 54 |
| cholecystokinin (cck)- F | GACATAGTGGCCATATACAACAGC | 2 |
| cholecystokinin-R | GCGTTCGTTCGCTTTCTC | 2 |
| trypsin (try)- F | AGCTCTGCTGTCCCATTCC | 84 |
| trypsin-R | TGCCACAACTGGAGTGTCAT | 84 |
| chymotrypsin (chy) -F | GCTCCCAGTACTCCCAACAA | 40 |
| chymotrypsin_R | ACACTGCTCATTGGACAGCA | 40 |
| carboxypeptidase A (cpa) -F | GCGTGACACTGGTCGTTATG | 27 |
| carboxypeptidase A -R | TCACATGTGGGCTTGATCTG | 27 |
| hepcidin-F | AGCTCTGCTGTCCCATTCC | 144 |
| hepcidin-R | TGCCACAACTGGAGTGTCAT | 144 |


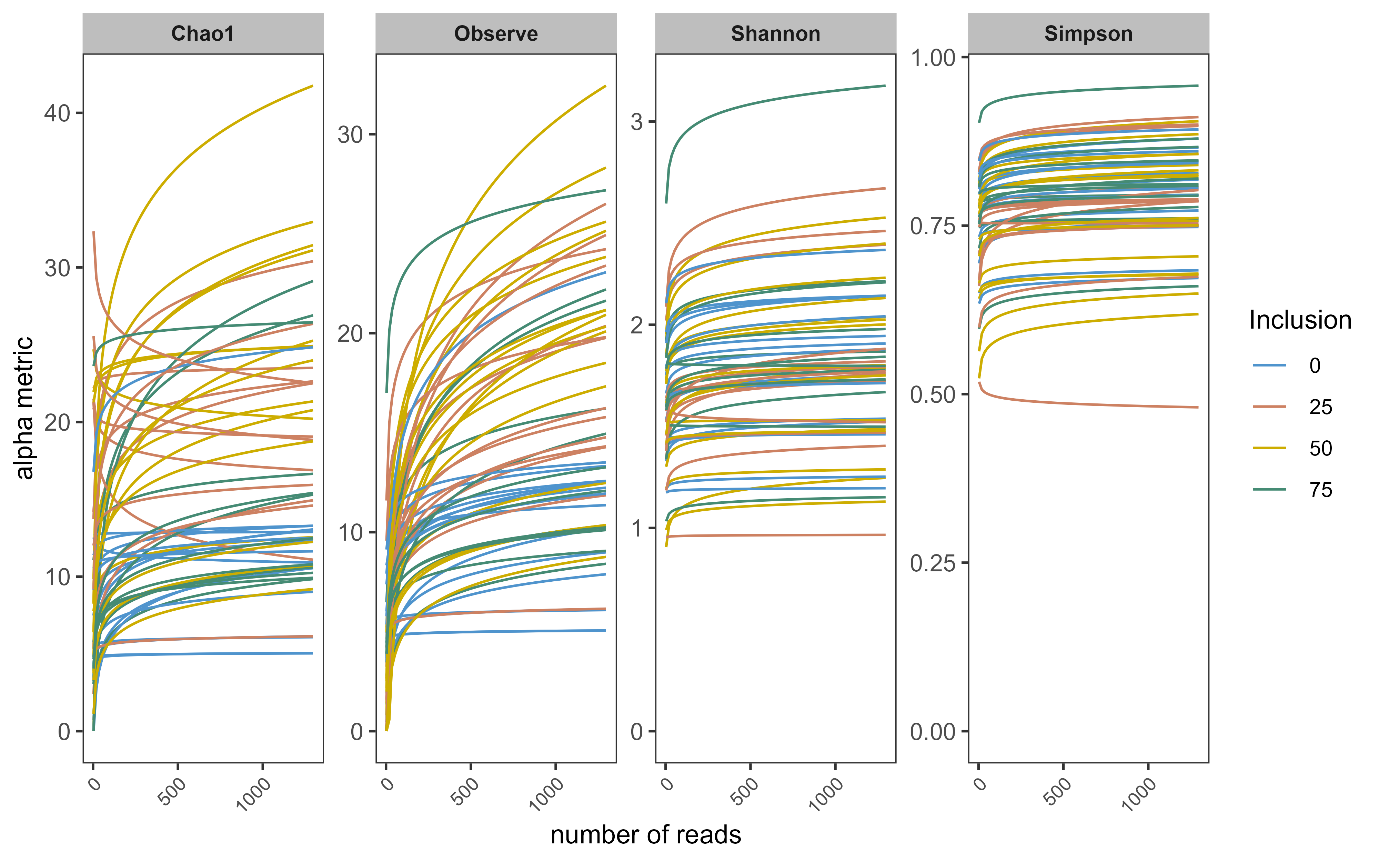


Fig. S1 Rarefaction curve indicating the depth and saturation level of samples with different SCP replacement levels. A stationary curve for bacterial diversity was observed for all samples except some for SCP 50.


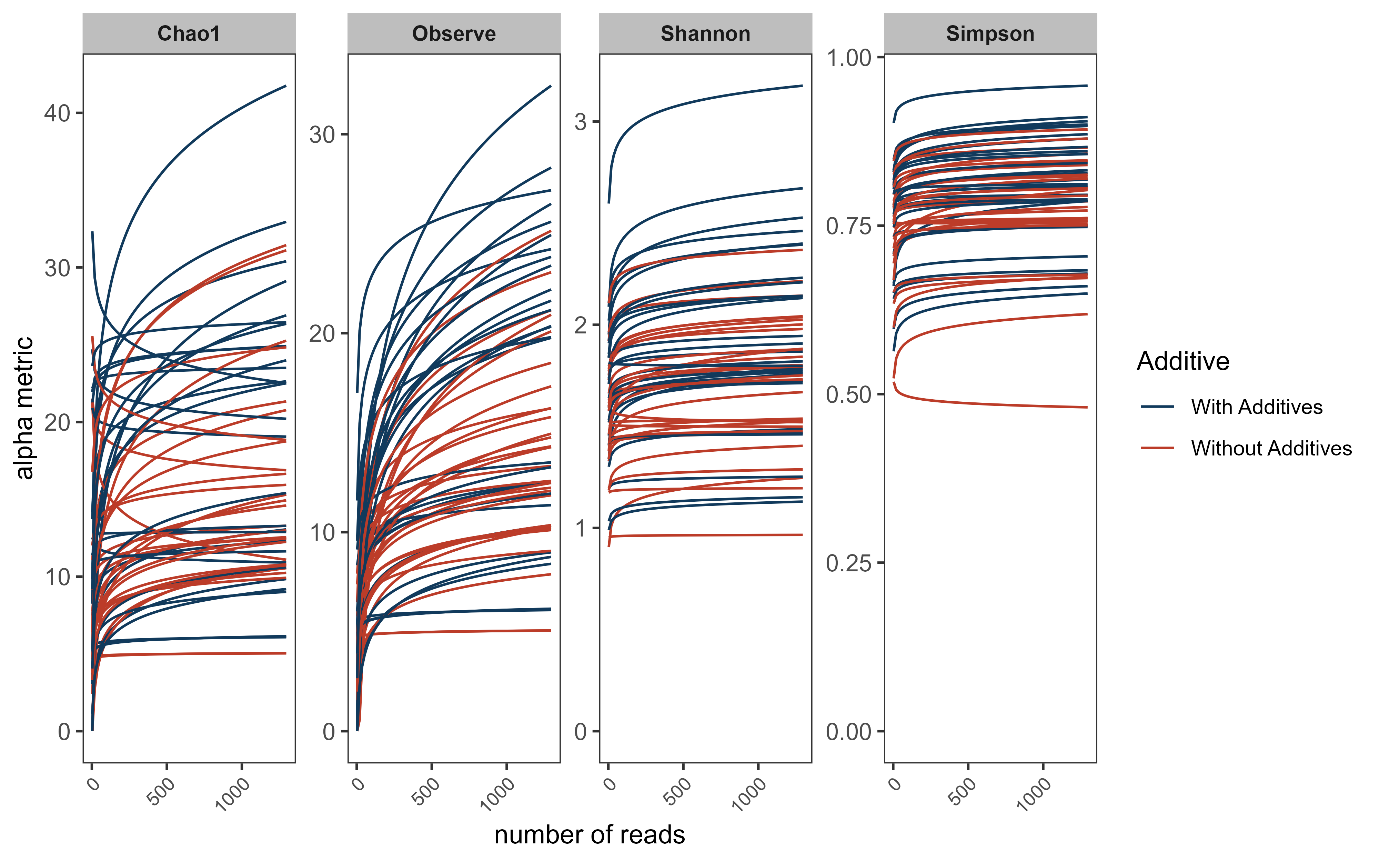


Fig. S2. Rarefaction curve indicating the depth and saturation level of samples with or without additives. A stationary curve for bacterial diversity was observed for all samples except for some samples with additives.


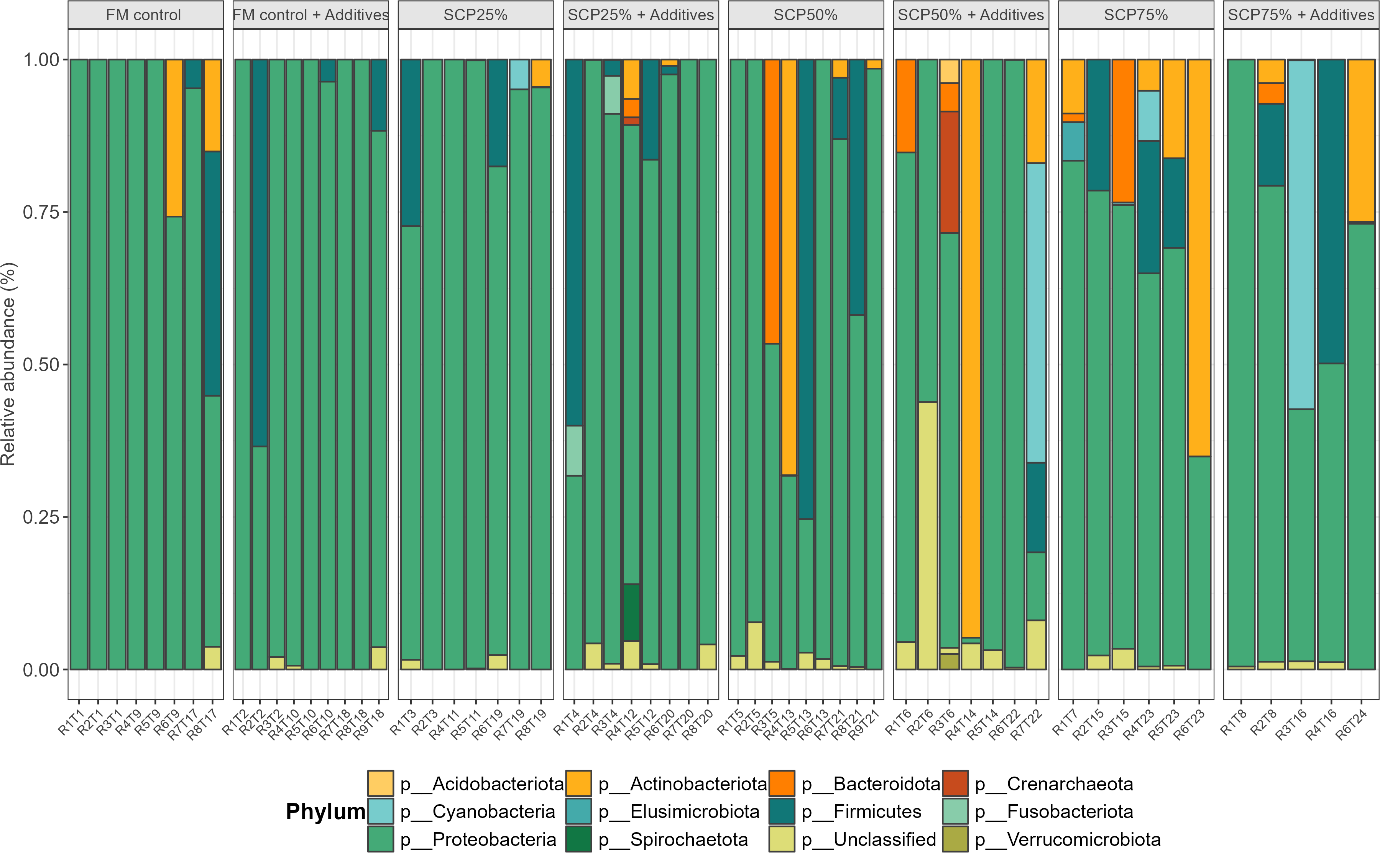


Fig. S3. Replicate-based relative abundance of Kingfish gut bacteria at phylum level in different treatments after feeding trial.
